# Revealing hyperactivated IFN-**γ** pathways in perianal fistulizing Crohn’s disease using single-cell and spatial multi-omics

**DOI:** 10.1172/JCI193413

**Published:** 2025-07-03

**Authors:** Siyan Cao, Khai M. Nguyen, Kaiming Ma, Tingyi Tan, Xin Yao, Ta-Chiang Liu, Malek Ayoub, Jalpa Devi, Sami Samaan, Yizhou Liu, Radhika Smith, Matthew L. Silviera, Steven R. Hunt, Paul E. Wise, Matthew G. Mutch, Sean C. Glasgow, William C. Chapman, Michelle L. Cowan, Matthew A. Ciorba, Marco Colonna, Parakkal Deepak, SPARC IBD Investigators

**Affiliations:** 1 Division of Gastroenterology,; 2 Department of Pathology and Immunology, and; 3 Section of Colon and Rectal Surgery, Washington University School of Medicine, St. Louis, Missouri, USA.; 4 The SPARC IBD Investigators are detailed in Supplemental Acknowledgments.

**Keywords:** Gastroenterology, Immunology, Cellular immune response, Inflammatory bowel disease, Therapeutics

## Abstract

Up to 40% patients with Crohn’s disease suffer from perianal disease, a debilitating complication with unclear etiology. This study identified unique pathophysiology which may help develop novel therapies.

**To the Editor:** Perianal fistulizing Crohn’s disease (PCD) occurs in up to 40% of patients with Crohn’s disease (CD), who are affected by substantial treatment resistance and high morbidity. The etiology of PCD remains poorly understood, hindering the development of preclinical models and effective therapies (1). Here, we utilized a multi-omics approach, including single-cell RNA sequencing (scRNA-Seq), cytometry by TOF (CyTOF), and spatial transcriptomics (ST) to characterize PCD, CD without perianal disease (NPCD), and idiopathic/cryptoglandular perianal fistula (IPF).

Fistula tract biopsies of 9 individuals with PCD and 6 with IPF (Supplemental Table 1; supplemental material available online with this article; https://doi.org/10.1172/JCI193413DS1) were analyzed using scRNA-Seq, which generated 56,560 high-quality cells (Figure 1A and Supplemental Figure 1A). Single-cell pathway analysis (SCPA) identified IFN-γ and TNF-α signaling as top activated pathways in PCD versus IPF (Figure 1B). Hyperactivated IFN-γ–responsive genes, including JAKs and STAT1, were present in major cell populations from PCD fistulas (Figure 1C and Supplemental Figure 1B). The transcriptomic data were validated by IHC, which showed increased IFN-γ and phosphorylated STAT1 in PCD (Figure 1D). By reanalyzing published scRNA-Seq datasets of rectum (2) (active PCD vs. inactive/healed PCD; *n* = 6/group) and colon and terminal ileum (3) (PCD vs. NPCD; *n* = 8 for PCD, *n* = 39 for NPCD), we showed upregulated IFN-γ and TNF-α pathways in all the 3 locations in PCD intestine compared with NPCD (Figure 1E). Reclustering of the rectal cells (2) uncovered similar induction of IFN-γ–responsive genes in multiple epithelial and immune cells from individuals with PCD and from individuals with healed PCD (Supplemental Figure 1C). Thus, PCD is characterized by heightened IFN-γ response in both fistula tracts and intestinal mucosa. Analysis of rectal epithelial cells (2) identified enriched clusters 9 and 12 with elevated IFN-γ, TNF-α, and EMT pathways in PCD compared with NPCD. IFN-γ and TNF-α responses were significantly correlated with EMT scores (Figure 1F). Compared with IPF, PCD fistulas exhibited increased extracellular matrix organization by stromal cells and cell adhesions by endothelial cells (Supplemental Figure 1D).

Unbiased ligand-receptor analysis revealed PCD-enriched pairs (Supplemental Figure 2A) known to drive inflammation and EMT. Moreover, IFN-γ senders Th17 cells and Tregs were significantly enriched in PCD (Figure 1G). IFN-γ–producing Th17 cells represent a pathogenic antigen-induced state (4) (pathogenic Th17 [pTh17]), which may underlie the activated IFN-γ response. PCD myeloid cells overexpressed NLRP3, AREG, and IFN-γ–induced CXCL9/10 (Supplemental Figure 2B). Signaling by bacterial molecule LPS was upregulated in NLRP3+AREG+ cells (or LPS myeloid cells) and the entire myeloid compartment in PCD (Supplemental Figure 2C). Ligand-receptor analysis further revealed multiple myeloid-derived chemokines/cytokines that activate Th17 cells (Supplemental Figure 2D). IFN-γ–producing cells may also be upregulated by higher IL15 in PCD (Supplemental Figure 2E). Other elevated pathways in PCD myeloid cells include NOD2/RIPK2 and TL1A/DR3 (Supplemental Figure 2, G–I). Additionally, our CyTOF data revealed features of IFN-γ signaling and established LPS myeloid cells as a hallmark of PCD microenvironment across fistula tracts, fistula opening, and rectum (Supplemental Figure 2, J–L).

Anti-TNFs currently have the best evidence for PCD treatment, with unclear mechanisms (1). We subdivided our scRNA-Seq PCD cohort into anti-TNF–treated or anti-TNF–naive groups based on treatment at sampling (*n* = 4/group; all patients on anti-TNFs later responded to therapy). Weighted gene coexpression network analysis identified 2 gene modules significantly correlated with anti-TNFs: the “lightpink1” module suppressed by anti-TNFs associated with immune cell activation and IFN response, while the “royalblue2” module induced by anti-TNFs supported cell proliferation and wound healing (Supplemental Figure 2, M–O).

ST of IPF and PCD fistula tracts (*n* = 3/group) generated 6 spatially correlated clusters (C0–5; Supplemental Figure 3A). Epithelial cells lining the fistula tract in C1 primarily mapped to IFNGR+TNFR+, IFNG/TNF-responsive colonocytes (Supplemental Figure 3B), further supporting the roles of IFN-γ and TNF-α signaling in fistulization. LPS myeloid cells were ubiquitously present at tract-adjacent spots, suggesting their interactions with microbial elements in the fistula tract (Supplemental Figure 3C), while pTh17 closely colocalized with LPS myeloid cells (Supplemental Figure 3D). Notably, these cells were present in all PCD samples but were scarce in IPF (Supplemental Figure 3E).

Finally, we validated our single-cell findings using intestinal bulk RNA-Seq data of intestinal samples from patients with active PCD (*n* = 12), inactive PCD (*n* = 23), and NPCD (*n* = 84) in an independent SPARC-IBD cohort. The analysis demonstrated hyperactivation of IFN-γ response (e.g., STAT1, IRF1; Figure 1H), EMT/tissue remodeling, inflammation, and endoplasmic reticulum stress in PCD tissues (Supplemental Figure 3F). Current PCD treatments (e.g., anti-TNFs and upadacitinib) are mainly approved through registrational trials in luminal CD; they have moderate efficacy and cannot heal the fistulas in most cases (1, 5). The therapeutic potential of IFN-γ antagonists warrants investigation using physiologically relevant PCD models.

For detailed methods, information regarding sex as a biological variable, statistics, study approval, author contributions, data availability, and acknowledgments, see the supplemental materials.

## Supplementary Material

Supplemental data

Supplemental table 1

Supporting data values

## Figures and Tables

**Figure 1:**
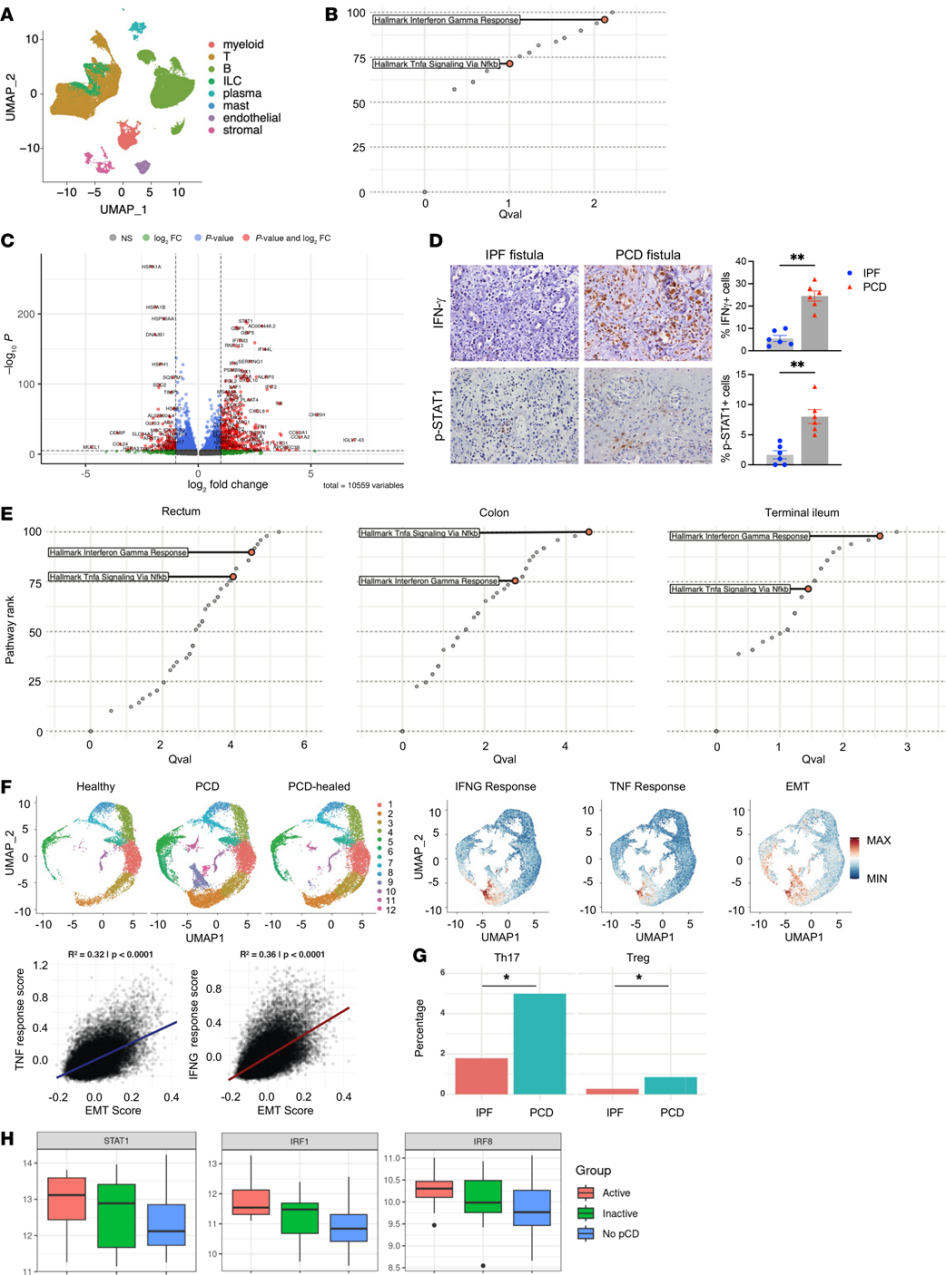
Hyperactivated IFN-γ signaling is a distinguishing feature of PCD in both fistula tracts and intestinal mucosa. (**A**) UMAP of major cell compartments in perianal fistulas. (**B**) Top upregulated pathways in PCD versus IPF fistulas by SCPA. (**C**) Altered gene expression in monocytes, macrophages, and dendritic cells in PCD versus IPF fistulas. (**D**) Representative IHC images and quantification in fistula tracts. Scale bar: 50 μm. **P < 0.01. (**E**) Top upregulated pathways in rectum (PCD vs. PCD healed) and colon/terminal ileum (PCD vs. NPCD) by SCPA. (**F**) UMAP of rectal epithelial cells; single-cell module scores; and correlation between TNF or IFNG response and EMT scores. (**G**) IFN-γ senders in PCD and IPF. (**H**) IFN-γ downstream genes in bulk RNA-Seq of CD intestinal samples. *P* values generated by Dunn’s post hoc test: STAT1, 0.019 (active vs. no PCD); IRF1, 0.00024 (active vs. no PCD) and 0.012 (inactive vs. no PCD); IRF8, 0.014 (active vs. no PCD).

## Version 1 07/03/2025

In-Press Preview

## Version 2 09/02/2025

Electronic publication
