# Supplementary material for: Revealing hyperactivated IFN-γ pathways in perianal fistulizing Crohn’s disease using single-cell and spatial multi-omics
Source: J Clin Invest. 2025 Jul 3;135(17):e193413. doi: 10.1172/JCI193413 (PMC12404762; doi:10.1172/JCI193413)
Supplement: Supplemental data [file jci-135-193413-s143.pdf]

Hyperactivated Interferon-γ Pathways in Perianal Fistulizing Crohn's Disease by Single-Cell and Spatial Multi-omics

Siyan Cao[1,*,#], Khai M. Nguyen[2,*], Kaiming Ma[1], Tingyi Tan[1], Xin Yao[1], Ta-Chiang Liu[2], Malek Ayoub[1], Jalpa Devi[1], Sami Samaan[1], Yizhou Liu[2], Radhika Smith[3,4], Matthew Silviera[3], Steven R. Hunt[3], Paul E. Wise[3], Matthew G. Mutch[3], Sean C. Glasgow[3,5], William C. Chapman Jr.[3], Michelle Cowan[3], Matthew A. Ciorba[1], Marco Colonna[2,#], Parakkal Deepak[1,#]; SPARC-IBD Investigators.

[1]Division of Gastroenterology, Department of Medicine, Washington University School of Medicine, St. Louis, MO 63110.

[2]Department of Pathology and Immunology, Washington University School of Medicine, St. Louis, MO 63110.

[3]Section of Colon and Rectal Surgery, Division of General Surgery, Department of Surgery, Washington University School of Medicine, St. Louis, MO 63110.

[4]Current address: AdventHealth Medical Group Colorectal Surgery at Winter Park, Winter Park, FL 32792.

[5]Current address: James H. Quillen VA Medical Center, Johnson City, TN 37604.

*Authors share co-first authorship.

#Authors share co-senior authorship.

Address correspondence to: Siyan Cao, email: caos@wustl.edu, Marco Colonna, email: mcolonna@wustl.edu, Parakkal Deepak, email: deepak.parakkal@wustl.edu

Conflict-of-interest statement

Marco Colonna, MD received research grants from Pfizer and Aclaris Therapeutics, unrelated to the data in the study. Matthew Ciorba, MD has received grants unrelated to the current content

from AbbVie, Takeda, Pfizer, and Janssen. Parakkal Deepak, MBBS MS received research support under a sponsored research agreement unrelated to the data in the study and/or consulting from Johnson and Johnson, Pfizer, AbbVie, Arena Pharmaceuticals, Bristol Myers Squibb, CorEvitas LLC, Sandoz, Takeda Pharmaceuticals, Direct Biologics, Prometheus Biosciences, Lilly, Teva Pharmaceuticals, Merck, ExeGI Pharmaceuticals, AGMB, Landos Pharmaceuticals, Tr1X, and Boehringer Ingelheim. No conflict-of-interest is related to this study.

Supplemental Methods

Sex as a biological variable

Both male and female sexes were involved in this study. Sex was not considered as a biological variable.

Patient Recruitment and Sample Collection

We recruited patients with a history of: 1) PCD (n = 24) confirmed by magnetic resonance imaging of the pelvis and/or examination under anesthesia (EUA) by a colorectal surgeon; 2) NPCD (n = 29); 3) IPF without a history of IBD, diagnosed on EUA by a colorectal surgeon (n=10; **Supplemental Table 1**). Exclusion criteria include prior diagnosis of infection- or malignancy-related secondary perianal fistulas, active infection or malignancy involving the anorectal area, contraindication to biopsies, patients under 18 years of age, pregnancy, or those unable to provide informed consent. Three biopsies each were taken using endoscopic biopsy forceps from the fistula tracts, external opening of the fistula, and/or nearby rectal mucosa (near internal fistula opening) from patients in PCD and IPF groups during routine EUA. Random rectal biopsies were taken from NPCD patients during routine colonoscopy. For each location sampled, biopsies were also collected for routine pathology assessment by two GI pathologists. Samples were cryopreserved within one hour of collection as previously described(1). Formalin-fixed paraffin-embedded blocks of fistula tracts from 6 patients with PCD were obtained from the Division of Anatomic & Molecular Pathology at Washington University School of Medicine. Patients' response after tissue collection was assessed based on clinical symptoms, EUA, and/or MRI.

The biopsies were kept in complete RPMI medium on ice immediately after collection and transported to the lab, where they were changed into a freezing medium (10% dimethyl sulfoxide in fetal calf serum), transferred to a pre-chilled Mr. Frosty™ (ThermoFisher) container with isopropanol, and kept in -80°C. The samples were then transferred to liquid nitrogen 24h later for storage. The cryopreservation of samples, isolation of immune cells, and staining for CyTOF

followed the sample protocols we recently described(2). Briefly, the biopsies were kept in complete RPMI medium on ice after collection and immediately transported to the lab, where they were changed into a freezing medium with 10% dimethyl sulfoxide, frozen in -80°C in a freezing container and transferred to liquid nitrogen 24h later. To minimize variation, the samples were processed in batches as described before. Briefly, the cryopreserved specimens were gently thawed in 37°C water bath, mucosal immune cells were extracted by digesting tissues in complete RPMI medium containing collagenase IV at 37°C for 50 min under agitation. Cells were then filtered and subjected to density gradient centrifugation using Percoll solutions.

CyTOF

Cells were processed for CyTOF as we previously described(1). Cells were washed with Cy-FACS buffer (CyPBS, Rockland, MB-008; 0.1% BSA, Sigma, A3059; 0.02% Sodium Azide, Sigma, 71289, 2mM EDTA, Hoefer, GR123-100) and stained with antibodies on ice for an hour. After two washes cells were stained with cisplatin (Enzo Life Sciences, NC0503617) for one minute to label dead cells. They were then washed twice, fixed in 4% PFA (Electron Microscopy Sciences, 15710) for 15 min, spun down and stained with barcodes for 30 min. The cells were then re-suspended in Intercalator-Ir125 (Fluidigm, 201192 A) overnight. Cells were washed, counted, and analyzed using CyTOF2/Helios instrument (Fluidigm) as previously described(1).
Samples were manually gated using Cytobank as described to exclude background, dead cells (Cisplatin+), doublets (DNA1/2 stain), and normalization beads. Dimensionality reduction was performed on CD45+ CD3+ CD19- T cells, CD45+ CD3- CD19+ B cells, and CD45+ CD3- CD19- innate immune cells to identify cell clusters based on surface markers, using the viSNE tool in Cytobank. Circulating Th17s were defined as CD103- CD45RO+ CD161+ CD127+ CD4+ T cells, as in (2). CD172a/b+ TREM1+ MNPs were defined as CD14+ CD33+ CD172a/b+ TREM1+ innate immune cells. Tc17 cells were defined as CD26+ CD161+ CD8+ T cells. Cell populations

expressing certain markers (e.g. CD39+ CD4+) were calculated by summing counts of clusters expressing these proteins.

Library preparation and sequencing

scRNA-seq library was prepared using the 10X Chromium Next GEM Single Cell 5' Kit v2, Chromium Single Cell Human TCR Amplification Kit, and Chromium Single Cell Human BCR Amplification Kit, respectively. Spatial transcriptomic library was prepared using the 10X Visium V4 – FFPE V2 chemistry. Sequencing was performed on a NovaSeq X Plus sequencer.

scRNA-seq data preprocessing, quality control, and cluster annotation

scRNA-seq data was aligned to the hg38 genome build and processed using the CellRanger suite (v7.1.1), and count matrices were loaded into Seurat (v4.4.0)(3). Cells with < 1000 or > 4000 unique detected features, > 30000 transcripts, and > 10% percent mitochondrial genes were discarded. Doublets were removed using DoubletFinder(4). Normalization was done using SCTransform(5), and batch correction was performed using the reciprocal PCA approach in Seurat. Differential expression testing was done using the Wilcoxon Rank Sum test.

Following dimensionality reduction and initial clustering, coarse cell types were identified as follows: B cells (MS4A1), plasma cells (SDC1), T cells (CD3D), ILCs (CD3D- cells that clustered closely to T cells), mononuclear phagocytes (ITGAX), plasmacytoid dendritic cells (pDCs; CLEC4C), mast cells (KIT, TPSAB1, TPSB2), endothelial cells (VWF), stromal cells (TAGLN). Subclustering analysis was performed by re-clustering B and plasma cells, T cells and ILCs, myeloid cells (mononuclear phagocytes, pDCs and mast cells), and stromal cells.

Subclusters were annotated as follows. <u>T and innate lymphoid cells (ILC):</u> naïve CD4 (CD4+, CCR7+), regulatory T cells (Treg; FOXP3+ CTLA4+), T-helper 17 cells (Th17; CD4+ IL17A+ RORC+), follicular helper T cells (Tfh; CD4+ PDCD1+), mucosal-associated invariant T cells (MAIT; TRAV1-2+ KLRB1+ SLC4A10+), effector memory CD8 T cells (CD8 Tem; CD8+

CX3CR1+ GZMH+), resident memory CD8 T cells (CD8 Trm; CD8+ ITGAE+ CD69+), Trm precursor cells(6) (CD8+ ZNF683+), group 3 ILCs (ILC3; CD3D- KIT+ PCDH9+ IL23R+ RORC+), CD56^{hi} NK cells (CD3D- EOMES+ NCAM1^{high}), and CD56^{low} NK cells (CD3D- EOMES+ NCAM1^{low}). B cells: naïve B cells (IGHM+ IGHD+) and memory B cells (CD27+ CD86+ CD80+ TFRC+). Myeloid cells: monocytes (CD14+ FCGR3A+ S100A8+ VCAN+), macrophages (C1QA+ C1QC+ HLA-DRA+), conventional dendritic cells (cDC; CLEC10A+ CLEC9A+ CD1E+ BATF3+), plasmacytoid dendritic cells (pDC; CLEC4C+ CD4+ IL3RA+), mature DC enriched in immunoregulatory molecules(7) (mRegDCs; CCR7+ CD40+ PDCD1LG2+ CD200+), and mast cells (KIT+ TPSB2+ TPSAB1+). Stromal cells: smooth muscle cells (ACTA2+ MYL9+ TAGLN+), inflammatory fibroblast-like cells (FAP+ MMP2+ MMP14+), PDGFRA^{hi} fibroblasts (PDGFRA^{hi}), DPT+ PI16+ fibroblasts (DPT+ PI16+), and myofibroblasts (COL4A1+ COL4A2+ MYO1B+ MYH11+).

Spatial transcriptomics analysis

Spatial transcriptomics (ST) data was aligned to the hg38 genome build and processed using Space Ranger (v3.0.1). Data was analyzed using Seurat (v4.4.0). Count data from individual samples were loaded and normalized using SCTransform(5). Normalized data was then merged into a single Seurat object and integrated using the canonical correlation analysis (CCA) method in Seurat. Cluster marker genes were identified using the FindConservedMarkers function and the Wilcoxon Rank Sum test.

Reanalysis of published datasets from Washburn et al.(8) and Kong et al.(9)

Washburn et al. dataset: Data was loaded into Seurat, and quality control was performed as described by the authors. Data was normalized using log transformation and batch-corrected using Harmony(10). Ambient RNA contamination was corrected using SoupX(11), and technical dropouts were imputed using adaptively thresholded low-rank approximation(12). Non-epithelial

cell clusters were annotated using Seurat's label transfer, with our in-house dataset as reference. Epithelial cells were identified based on KRT8 expression. Epithelial cell subclusters (as in Supplementary Fig 3) were annotated as follows: Colonocytes (C0, 2), IFNGR+ TNFR+ colonocytes (C1), cycling (C3, 7, 8), Goblet cells (C4, 5), crypt cells (C6), IFN-responsive (C9), BEST4+ epithelial cells (C10), TNF-responsive (C12).

Kong et al. dataset: Clinical information including the CD donors' perianal disease was kindly provided by Dr. Ashwin N. Ananthakrishnan at Massachusetts General Hospital. Data was loaded into Seurat and quality control was performed as described by the authors. Data was normalized using log transformation and batch-corrected with Harmony.

Pathway analysis, module scoring, and transcription factory activity inference.

Pathway analysis was performed via the SCPA R package(13), using the Hallmark human gene sets. Single-cell module scores were calculated using the Mann-Whitney U statistic(14). Interferon-g signaling, TNF-a signaling, and epithelial-mesenchymal transition scores (as in Figure 3) were calculated using the GOBP_RESPONSE_TO_TYPE_II_INTERFERON, GOBP_TUMOR_NECROSIS_FACTOR_MEDIATED_SIGNALING_PATHWAY, and GOBP_EPITHELIAL_TO_MESENCHYMAL_TRANSITION gene sets from MsigDB(15), respectively. Pathogenic Th17 (pTh17) signature gene module (as in Figure 7) consisted of CD3D, IL17A, and IFNG, while the LPS_myeloid signature gene module consisted of CD14, FCGR3A (encoding CD16), NLRP3, AREG, and TREM1. Transcription factor activity was inferred using the Python implementation of SCENIC (pySCENIC(16)).

Ligand-receptor signaling analysis

Ligand-receptor (LR) signaling analysis was performed using NICHES(17). Prior to analysis, normalized and annotated scRNA-seq/ST data underwent imputation using adaptively thresholded low-rank approximation(12) to correct for technical dropouts. NICHES was ran using

the fantom5 ligand-receptor database. Cluster marker LR pairs were identified using the receiver operating characteristic (ROC) test within Seurat's FindMarkers function, using an AUC threshold of 0.8.

Weighted gene co-expression network analysis

IPF samples were excluded from the scRNA-seq dataset, as was one PCD sample from a patient receiving Ustekinumab, resulting in a total of 8 samples (4/group). Raw scRNA-seq counts were pseudobulked using the summation method, and genes that were unannotated or encoding lncRNA were excluded. Pseudobulked matrix was then batch-corrected using ComBat_seq(18) with anti-TNF treatment status as a covariate. Adequate batch correction was confirmed using PCA. The matrix was then normalized using the regularized log transformation method in DESeq2(19), and genes with low variance were removed. The adjacency matrix was constructed using softPower of 9. Following construction of module eigengenes, modules with less than 25% dissimilarity were merged, resulting in a total of 32 modules.

Cell type deconvolution for ST

Spot deconvolution from ST data was performed using CARD(20). Genes with under 100 counts and spots with under 5 counts were excluded from deconvolution, and deconvolution was performed on each spatial sample separately. The single-cell reference was generated by combining our fistula scRNA-seq data and Washburn et al(8) rectal scRNA-seq dataset, to account for the lack of epithelial cells in our dataset. Briefly, the rectal dataset underwent SCTransform for consistency with our in-house data; the datasets were then integrated in Seurat using reciprocal PCA. A simplified cell annotation was used: Tfh, Treg, Th17, and naïve CD4/Tcm were grouped together as CD4 T cells; CD8 Trm precursor, CD8 Trm, CD8 Tem, MAIT were grouped together as CD8 T cells; CD56dim NK, CD56hi NK, ILC3 were grouped together as ILCs; naïve and memory B cells were grouped together as B cells; cDC1/2 and mRegDCs were grouped

together as DCs; and inflammatory-fibroblast-like, PDGFRAhi fibroblasts, and DPT+PI16+ fibroblasts were grouped together as fibroblasts. Raw counts were inputted for CARD deconvolution.

Bulk RNA-seq analysis

The bulk RNA-seq data were obtained from the Study of a Prospective Adult Research Cohort with IBD(21). For each patient in the cohort, the earliest available RNA-seq biopsy sample was selected. Perianal disease status was determined by matching clinical visit dates to biopsy collection dates within a +/- 3-month window. Patients were categorized into three groups: Active PCD, Inactive PCD, and No PCD (those with CD but with no past or current evidence for perianal CD). Raw gene-level count data were loaded. Metadata for selected patients were compiled, and technical replicates were collapsed based on unique patient identifiers. A DESeq2 object was constructed with sample group information as the design formula. Variance-stabilizing transformation (VST) was applied to normalized counts to limit the dependence of variance on mean gene expression. The expression of selected genes across the three groups was visualized using boxplots. Statistical significance was assessed using the Kruskal–Wallis test, followed by a post-hoc Dunn's test for pairwise comparisons. For the pathway analysis, genes significantly upregulated or downregulated in the pooled pCD (Active + Inactive) vs. No pCD comparison were selected. Biological process enrichment was conducted, and the results were visualized using dot plots.

Statistics

GraphPad Prism (GraphPad software, La Jolla, CA) was used for statistical analysis. Mann-Whitney test or Kruskal-Wallis test was used for the analysis of differences between 2 groups or more than 2 groups, respectively. Dunn test was performed for post-hoc analysis. P value < 0.05 was considered statistically significant.

Study approval

This study has been approved by the Institutional Review Board of Washington University School of Medicine (IRB ID #: 202107119).

Data availability

We have made data generated in this study, analytic methods, and study materials available to other researchers. The single-cell transcriptomics data has been submitted to NCBI's GEO (GSE277387).

Supporting data values were provided in the Supporting Data Values file.

The bulk RNA-seq data are from the Study of a Prospective Adult Research Cohort with IBD (SPARC-IBD), a component of the Crohn’s & Colitis Foundation’s IBD Plexus data exchange platform. SPARC IBD enrolls patients with an established or new diagnosis of IBD from sites throughout the United States and links data collected from the electronic health record and study specific case report forms. Patients also provide blood, stool and biopsy samples at selected times during follow-up. The design and implementation of the SPARC IBD cohort has been previously described(21). The SPARC IBD data are available upon approved application to Crohn’s & Colitis Foundation IBD Plexus (https://www.crohnscolitisfoundation.org/ibd plexus).

Author Contributions:

Conceptualization: SC, PD.

Data curation: SC, KMN (equal).

Formal analysis: SC, KMN (equal).

Funding acquisition: SC, PD.

Investigation: SC, KMN.

Methodology: SC, KMN, PD (equal).

Project administration: SC, PD.

Resources: SC, KM, TT, XY, T-CL, MA, JP, SS, YL, RS, MS, SRH, PEW, MGM, SCG, WCC, MC, MAC, PD.

Supervision: SC, PD.

Writing – original draft: SC, KMN, PD (equal).

Writing – review and editing: SC, KMN, PD, MC.

SC is first of the first authors because he conceptualized, designed, initiated, and supervised the study and played a major role in the data curation and analysis. KMN is the second of the first authors because he played a major role in the data curation and analysis.

Acknowledgements

The work was made possible by the generous supports of the American Gastroenterological Association (AGA) Fellowship-to-Faculty Transition Award (AGA2023-32-03, Siyan Cao), Digestive Disease Research Core Center (DDRCC) Pilot and Feasibility Award (P30 DK052574, Siyan Cao), NIH/NIDDK K08 Clinical Investigator Award (1K08DK140612, Siyan Cao), the NIH CTSA Grant UL1TR002345, and Washington University (WU) DDRCC (NIDDK P30 DK052574). The authors thank the stellar clinical research coordinators: Darren Billy Nix, Donald Jones, Ginny Van Teslaar, Vicki Martin, and Guadalupe Oliva Escudero at WU Inflammatory Bowel Disease Center for help with IRB, recruitment of patients, and sample collection. We thank the WU Genome Access Technology Center and Immunomonitoring Laboratory for help with scRNA-seq/spatial transcriptomics and CyTOF, respectively. Siyan Cao was also supported by a Crohn's & Colitis Foundation (CCF) Career Development Award, WU Clinical and Translational Research Funding Program, Lawrence C. Pakula, MD IBD Education & Innovation Fund, Doris Duke COVID-19 Fund to Retain Clinical Scientists Program, WU Precision Health Innovation Award, AGA Pilot Research Award, and American College of Gastroenterology (ACG) Clinical Research

Award. Parakkal Deepak was supported by an ACG Junior Faculty Development Award, IBD Plexus of the CCF, and Leo & Carean Goss Crohn's Disease Research Fund. Marco Colonna was supported by NIH R01s DK126969 and DK132327. Matthew A. Ciorba was supported by Givin' It All For Guts Foundation. The work was also supported by philanthropic support from the Drury Hotels Company LLC through the Foundation for Barnes-Jewish Hospital.

The results published here are in whole or partly based on data from the Study of a Prospective Adult Research Cohort with IBD (SPARC IBD). SPARC IBD is a component of the Crohn's & Colitis Foundation's IBD Plexus data exchange platform. SPARC IBD enrolls patients with an established or new diagnosis of IBD from sites throughout the United States and links data collected from the electronic health record and study specific case report forms. Patients also provide blood, stool and biopsy samples at selected times during follow-up. The design and implementation of the SPARC IBD cohort has been previously described(21).

SPARC IBD Participating Sites and Local Principal Investigators

Site	Principal Investigator(s)
Baylor College of Medicine	Richa Shukla, MD
Baylor Scott & White	Themistocles Dassopoulos, MD
Brigham & Women's Hospital	Josh Korzenik, MD<br>Scott Snapper, MD, PhD
Indiana University	Satya Kudara, MD
Mayo Clinic	Laura Raffals, MD
Mayo Clinic Arizona	Manreet Kaur, MD
Medical College of Wisconsin	Poonam Beniwal-Patel, MD
Mercy Medical Center	Ray Cross, MD
NYU Langone Medical Center	David Hudesman, MD

Scripps Healthcare	Mazer Ally, MD<br>Gauree Konijeti, MD<br>Rebecca Matro, MD
University of Alabama	Kirk Russ, MD
University of Cincinnati	Kara De Felice, MD
University of Chicago	Joel Pekow, MD<br>Sushila Dalal, MD
University Gastroenterology	Sheldon Lidofsky, MD
University of Maryland	Lauren George, MD
University of Michigan	Shrinivas Bishu, MD
University of Pennsylvania	Meenakshi Bewtra, MD, MPH, PhD<br>James D Lewis, MD, MSCE
University of Pittsburgh	Richard Duerr, MD
University of Wisconsin	Sumona Saha, MD, MS<br>Freddy Caldera, DO, MS
Vanderbilt University	Elizabeth Scoville, MD, MSCI
Washington University	Parakkal Deepak, MBBS, MS

Comma delineated:

Richa Shukla, MD (Baylor College of Medicine), Themistocles Dassopoulos, MD (Baylor Scott & White), Josh Korzenik, MD (Brigham & Women's Hospital), Scott Snapper, MD, PhD (Brigham & Women's Hospital), Satya Kudara, MD (Indiana University), Laura Raffals, MD (Mayo Clinic), Manreet Kaur, MD (Mayo Clinic Arizona), Poonam Beniwal-Patel, MD (Medical College of Wisconsin), Ray Cross, MD (Mercy Medical Center), David Hudesman, MD (NYU Langone

Medical Center), Mazer Ally, MD (Scripps Healthcare), Gauree Konijeti, MD (Scripps Healthcare), Rebecca Matro, MD (Scripps Healthcare), Kirk Russ, MD (University of Alabama), Kara De Felice, MD (University of Cincinnati), Joel Pekow, MD (University of Chicago), Sushila Dalal, MD (University of Chicago), Sheldon Lidofsky, MD (University Gastroenterology), Lauren George, MD (University of Maryland), Shrinivas Bishu, MD (University of Michigan), Meenakshi Bewtra, MD, MPH, PhD (University of Pennsylvania), James D. Lewis, MD, MSCE (University of Pennsylvania), Richard Duerr, MD (University of Pittsburgh), Sumona Saha, MD, MS (University of Wisconsin), Freddy Caldera, DO, MS (University of Wisconsin), Elizabeth Scoville, MD, MSCI (Vanderbilt University), Parakkal Deepak, MBBS, MS (Washington University)

Bulleted list:

- Richa Shukla, MD (Baylor College of Medicine)
- Themistocles Dassopoulos, MD (Baylor Scott & White)
- Josh Korzenik, MD (Brigham & Women's Hospital)
- Scott Snapper, MD, PhD (Brigham & Women's Hospital)
- Satya Kudara, MD (Indiana University)
- Laura Raffals, MD (Mayo Clinic)
- Manreet Kaur, MD (Mayo Clinic Arizona)
- Poonam Beniwal-Patel, MD (Medical College of Wisconsin)
- Ray Cross, MD (Mercy Medical Center)
- David Hudesman, MD (NYU Langone Medical Center)
- Mazer Ally, MD (Scripps Healthcare)
- Gauree Konijeti, MD (Scripps Healthcare)

- Rebecca Matro, MD (Scripps Healthcare)
- Kirk Russ, MD (University of Alabama)
- Kara De Felice, MD (University of Cincinnati)
- Joel Pekow, MD (University of Chicago)
- Sushila Dalal, MD (University of Chicago)
- Sheldon Lidofsky, MD (University Gastroenterology)
- Lauren George, MD (University of Maryland)
- Shrinivas Bishu, MD (University of Michigan)
- Meenakshi Bewtra, MD, MPH, PhD (University of Pennsylvania)
- James D. Lewis, MD, MSCE (University of Pennsylvania)
- Richard Duerr, MD (University of Pittsburgh)
- Sumona Saha, MD, MS (University of Wisconsin)
- Freddy Caldera, DO, MS (University of Wisconsin)
- Elizabeth Scoville, MD, MSCI (Vanderbilt University)
- Parakkal Deepak, MBBS, MS (Washington University)

References

1. Cao S, Nguyen KM, Ma K, Du X, Liu X, Ulezko Antonova A, et al. Mucosal Single-Cell Profiling of Crohn's-Like Disease of the Pouch Reveals Unique Pathogenesis and Therapeutic Targets. *Gastroenterology*. 2024;167(7):1399-414 e2.
2. Jaeger N, Gamini R, Cella M, Schettini JL, Bugatti M, Zhao S, et al. Single-cell analyses of Crohn's disease tissues reveal intestinal intraepithelial T cells heterogeneity and altered subset distributions. *Nature communications*. 2021;12(1):1921.
3. Hao Y, Hao S, Andersen-Nissen E, Mauck WM, Zheng S, Butler A, et al. Integrated analysis of multimodal single-cell data. *Cell*. 2021;184(13):3573-87. e29.
4. McGinnis CS, Murrow LM, and Gartner ZJ. DoubletFinder: doublet detection in single-cell RNA sequencing data using artificial nearest neighbors. *Cell systems*. 2019;8(4):329-37. e4.
5. Hafemeister C, and Satija R. Normalization and variance stabilization of single-cell RNA-seq data using regularized negative binomial regression. *Genome biology*. 2019;20(1):296.
6. Parga-Vidal L, Behr FM, Kragten NA, Nota B, Wesselink TH, Kavazović I, et al. Hobit identifies tissue-resident memory T cell precursors that are regulated by Eomes. *Science immunology*. 2021;6(62):eabg3533.
7. Maier B, Leader AM, Chen ST, Tung N, Chang C, LeBerichel J, et al. A conserved dendritic-cell regulatory program limits antitumour immunity. *Nature*. 2020;580(7802):257-62.
8. Washburn S, Maddipatla SC, Murthy S, Dodd A, Pelia RS, Kolachala VL, et al. Persistent Inflammation of the Rectum in Perianal Fistulizing Crohn's Disease Is Associated With Goblet Cell Function. *Gastro Hep Advances*. 2024;3(1):131-3.
9. Kong L, Pokatayev V, Lefkovith A, Carter GT, Creasey EA, Krishna C, et al. The landscape of immune dysregulation in Crohn's disease revealed through single-cell transcriptomic profiling in the ileum and colon. *Immunity*. 2023;56(2):444-58 e5.
10. Korsunsky I, Millard N, Fan J, Slowikowski K, Zhang F, Wei K, et al. Fast, sensitive and accurate integration of single-cell data with Harmony. *Nature methods*. 2019;16(12):1289-96.
11. Young MD, and Behjati S. SoupX removes ambient RNA contamination from droplet-based single-cell RNA sequencing data. *Gigascience*. 2020;9(12):giaa151.
12. Linderman GC, Zhao J, Roulis M, Bielecki P, Flavell RA, Nadler B, et al. Zero-preserving imputation of single-cell RNA-seq data. *Nature communications*. 2022;13(1):192.
13. Bibby JA, Agarwal D, Freiwald T, Kunz N, Merle NS, West EE, et al. Systematic single-cell pathway analysis to characterize early T cell activation. *Cell Reports*. 2022;41(8).
14. Andreatta M, and Carmona SJ. UCell: Robust and scalable single-cell gene signature scoring. *Computational and structural biotechnology journal*. 2021;19:3796-8.
15. Liberzon A, Subramanian A, Pinchback R, Thorvaldsdóttir H, Tamayo P, and Mesirov JP. Molecular signatures database (MSigDB) 3.0. *Bioinformatics*. 2011;27(12):1739-40.

16. Van de Sande B, Flerin C, Davie K, De Waegeneer M, Hulselmans G, Aibar S, et al. A scalable SCENIC workflow for single-cell gene regulatory network analysis. *Nature protocols*. 2020;15(7):2247-76.
17. Raredon MSB, Yang J, Kothapalli N, Lewis W, Kaminski N, Niklason LE, et al. Comprehensive visualization of cell–cell interactions in single-cell and spatial transcriptomics with NICHES. *Bioinformatics*. 2023;39(1):btac775.
18. Zhang Y, Parmigiani G, and Johnson WE. ComBat-seq: batch effect adjustment for RNA-seq count data. *NAR genomics and bioinformatics*. 2020;2(3):lqaa078.
19. Love MI, Huber W, and Anders S. Moderated estimation of fold change and dispersion for RNA-seq data with DESeq2. *Genome biology*. 2014;15:1-21.
20. Ma Y, and Zhou X. Spatially informed cell-type deconvolution for spatial transcriptomics. *Nature biotechnology*. 2022;40(9):1349-59.
21. Raffals LE, Saha S, Bewtra M, Norris C, Dobes A, Heller C, et al. The Development and Initial Findings of A Study of a Prospective Adult Research Cohort with Inflammatory Bowel Disease (SPARC IBD). *Inflamm Bowel Dis*. 2022;28(2):192-9.

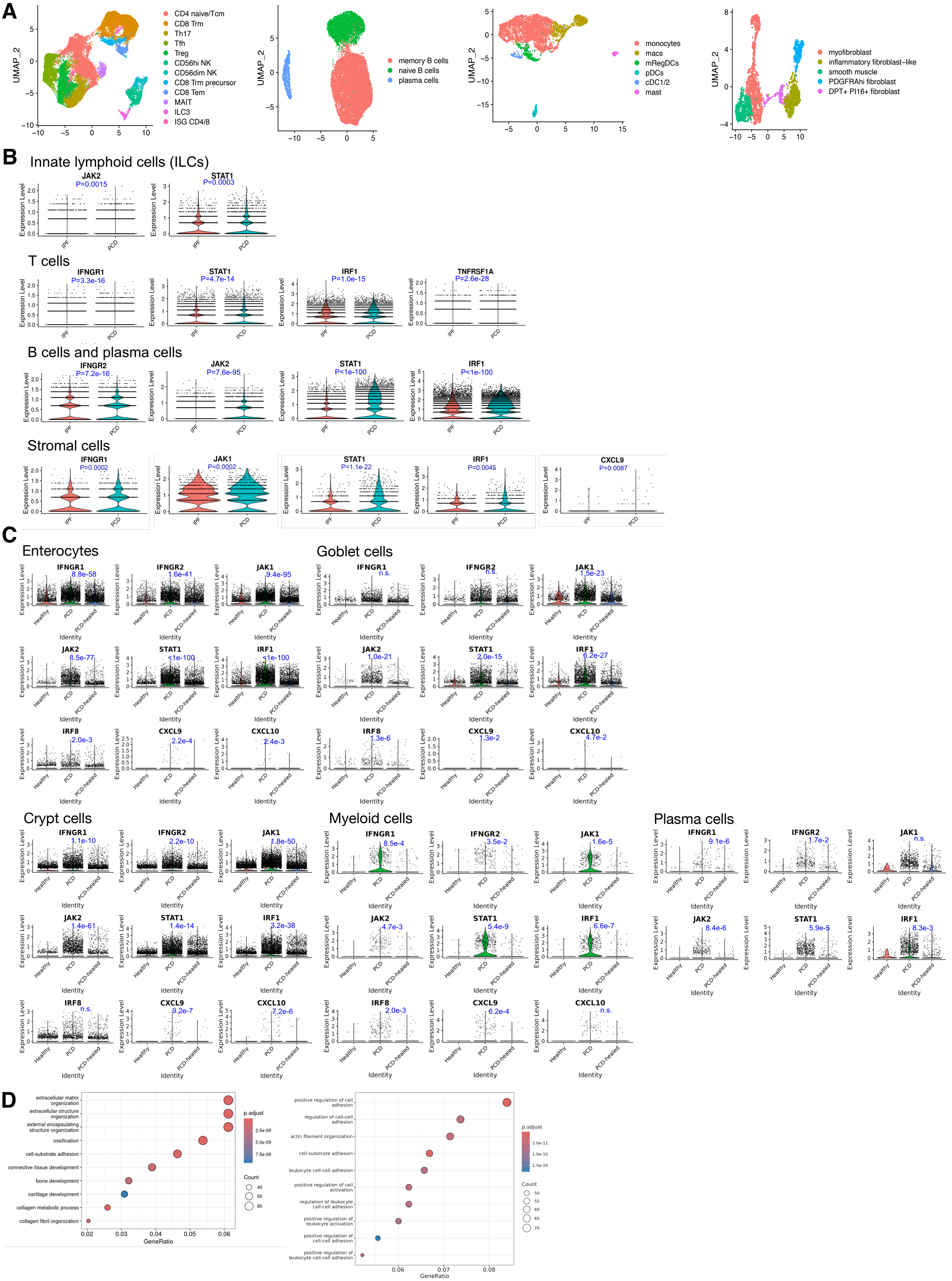

Supplementary Figure 1. (A) UMAP plots showing subsets of T cells and ILCs, B cells and plasma cells, myeloid cells, and stromal cells. (B) Increased single-cell expression of IFN-γ-related genes in PCD vs. IPF fistulas. (C) Increased single-cell expression of IFN-γ-related genes in the rectum of PCD vs. PCD-healed and healthy controls. P value for PCD vs. PCD-healed is shown on each plot. n.s., not significant. (D) Elevated pathways in stromal cells (left) and endothelial cells (right) in PCD vs. IPF fistulas.

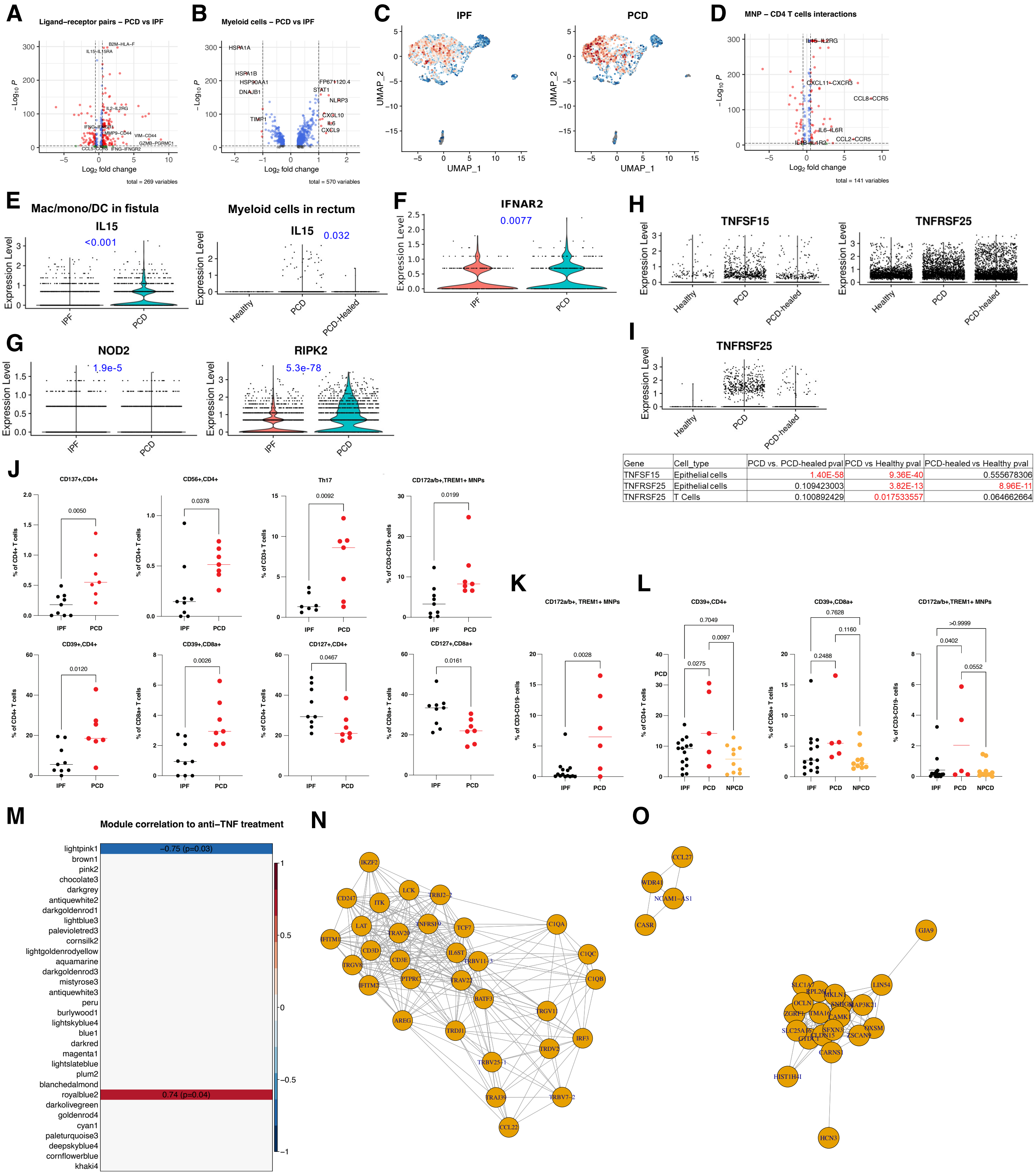

Gene	Cell_type	PCD vs. PCD-healed pval	PCD vs Healthy pval	PCD-healed vs Healthy pval
TNFSF15	Epithelial cells	1.40E-58	9.36E-40	0.555678306
TNFRSF25	Epithelial cells	0.109423003	3.82E-13	8.96E-11
TNFRSF25	T Cells	0.100892429	0.017533557	0.064662664

Supplementary Figure 2. (A-D) Myeloid cell-Th17 interactions underlie IFN-γ response in PCD fistulas by scRNA-seq. (A) Differentially activated ligand-receptor signaling pairs. (B) Differentially expressed genes between PCD and IPF myeloid cells. (C) UMAP plots of single-cell module scores for LPS signaling in myeloid cells. (D) Top enriched ligand-receptor signaling pairs between mononuclear phagocytes (MNP) and CD4 T cells in PCD. (E) Increased IL15 expression in PCD fistula (left) and PCD rectum (right). (F) Increased IFNAR2 expression in LPS_myeloid cells from PCD fistula tracts. (G) Elevated expression of NOD2 and RIPK2 in Mac/mom/DC in PCD fistulas. P value is shown on each plot. (H, I) Elevated expression of TNFSF15 (encoding TL1A) and TNFRSF25 (encoding DR3) in epithelial cells (H) and T cells (I) from PCD rectum. P values in the table below. (J-L) CyTOF data of cell frequencies in fistula tracts (J), external fistula opening (K), and rectum (L). (M-O) Gene expression modules in PCD fistula tracts correlated with anti-TNFs. (M) Co-expression gene modules identified by WGCNA. Significant correlations: red – positive correlation; blue – negative correlation. (N) Network of key lightpink1 module members associated with T and myeloid cell function. (O) Network of key royalblue2 module members associated with structural integrity, metabolism, proliferation, and immune homeostasis.

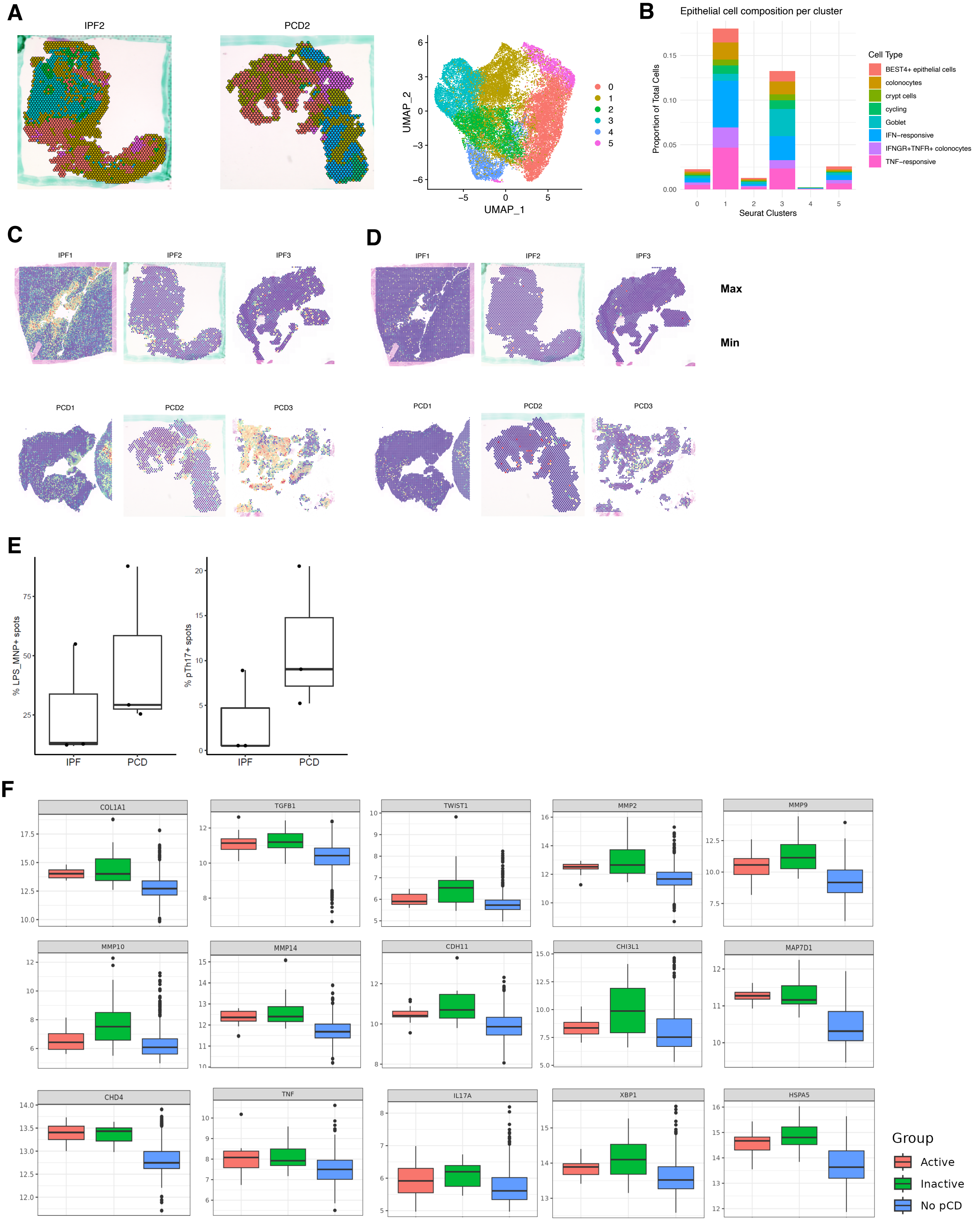

Supplementary Figure 3. (A-E) Spatial transcriptomics identified shared niches across fistula tracts. (A) Left/middle: representative plots showing spatial organization of each cluster in IPF and PCD fistulas; right: UMAP plot of spatial clusters. (B) Epithelial cell composition across spatial clusters. (C) Single-spot scores of LPS_myeloid gene module in IPF and PCD fistulas. (D) Single-spot scores of pTh17 gene module in IPF and PCD fistulas. (E) Percent of pTh17 and LPS_myeloid cells in IPF and PCD fistula samples based on spatial analysis. (F) Gene expression from bulk RNA-seq of intestinal samples of active PCD, inactive PCD, and CD without perianal disease. For all genes, p value < 0.05 for Active vs. No PCD; p value < 0.01 for Inactive vs. No PCD.
