# Supplementary material for: Revealing hyperactivated IFN-γ pathways in perianal fistulizing Crohn’s disease using single-cell and spatial multi-omics
Source: J Clin Invest. 2025 Jul 3;135(17):e193413. doi: 10.1172/JCI193413 (PMC12404762; doi:10.1172/JCI193413)
Supplement: Supplemental table 1 [file jci-135-193413-s144.pdf]

	Perianal fistulizing CD (PCD; N=24)	CD without perianal disease (NPCD; N=10)	Idiopathic/cryptoglandular fistula (IPF; N=29)	PCD vs. NPCD *P* value
**Age, mean (SD), y**	40.9 (11.0)	43.3 (11.1)	47.0 (14.6)	0.58
**Sex, N (%)**				0.33
Female	14 (58.3)	4 (40.0)	10 (34.5)	
Male	10 (41.7)	6 (60.0)	19 (65.5)	
**Race, N (%)**				0.79
Asian	1 (4.2)	0 (0.0)	1 (3.4)	
Black or African American	3 (12.5)	1 (10.0)	3 (10.3)	
Other Pacific Islander	0 (0.0)	0 (0.0)	0 (0.0)	
White	20 (83.3)	9 (90.0)	25 (86.2)	
**Tobacco Use, N (%)**				0.25
Current	7 (29.2)	1 (10.0)	6 (20.7)	
Former	4 (16.7)	4 (40.0)	7 (24.1)	
Never	13 (54.2)	5 (50.0)	16 (55.2)	
**Duration of Diagnosis, mean (SD), y**	16.2 (11.2)	16.3 (9.4)	4.6 (8.2)	0.98
**Age at diagnosis, N (%)**				0.70
A1 [<16 y]	3 (12.5)	2 (20.0)	0 (0.0)	
A2 [17-40 y]	20 (83.3)	8 (80.0)	15 (51.7)	
A3 [>40 y]	1 (4.2)	0 (0.0)	14 (48.3)	
**Location, N (%)**				0.05
L1	0 (0.0)	1 (10.0)	0 (0.0)	
L2	7 (29.2)	1 (10.0)	0 (0.0)	
L3	17 (70.8)	7 (70.0)	0 (0.0)	
L4	0 (0.0)	1 (10.0)	0 (0.0)	
None	0 (0.0)	0 (0.0)	29 (100.0)	
**Behavior, N (%)**				0.66
B1	8 (33.3)	3 (30.0)	0 (0.0)	
B2	6 (25.0)	4 (40.0)	0 (0.0)	
B3	10 (41.7)	3 (30.0)	0 (0.0)	
None	0 (0.0)	0 (0.0)	29 (100.0)	
**TOpClass Classification[#] at Time of Biopsy N (%)**				
Class 2a: Repair	17 (70.8%)	-	-	
Class 2b: Symptom control	3 (12.5%)			
Class 2cii: Gradually debilitating disease	3 (12.5%)			
Class 3: Severe disease with exhausted perineum	1 (4.2%)			
**Antidiarrheal, N (%)**				0.39
Yes	11 (45.8)	3 (30.0)	5 (17.2)	
No	13 (54.2)	7 (70.0)	24 (82.8)	
**5-Aminosalicylic Acid, N (%)**				0.76
Yes	6 (25.0)	3 (30.0)	0 (0.0)	
No	18 (75.0)	7 (70.0)	29 (100.0)	
**Biologics, N (%)**				0.95
Adalimumab	3 (12.0)	1 (10.0)	0 (0.0)	
Infliximab	4 (16.0)*	1 (10.0)	0 (0.0)	
Ustekinumab	6 (24.0)	3 (30.0)	0 (0.0)	
Vedolizumab	1 (4.0)	1 (10.0)	0 (0.0)	
None	11 (44.0)	4 (40.0)	29 (100.0)	
**Corticosteroids**				0.22
Yes	3 (12.5)	3 (30.0)	1 (3.4)	
No	21 (87.5)	7 (70.0)	28 (96.6)	
**Immunomodulators**				0.01
Yes	4 (16.7)	6 (60.0)	0 (0.0)	
No	20 (83.3)	4 (40.0)	29 (100.0)	
**Antibiotics**				0.01
Yes	4 (16.7)	6 (60.0)	7 (24.1)	
No	20 (83.3)	4 (40.0)	22 (75.9)	
**Proctitis Endoscopy**	5 (20.8)	2 (20.0)	0 (0.0)	0.96
**Abdominal Surgery**	10 (41.7)	4 (40.0)	0 (0.0)	0.93
**Proctitis Histology**	6 (25.0)	1 (10.0)	0 (0.0)	0.32

**Proctitis MRI**	3 (12.5)	1 (10.0)	1 (3.4)	0.84
**Sample Type[+]**				
CyTOF	7 (28.0)	10 (100.0)	15 (50.0)	
scRNA-seq	9 (36.0)	0 (0.0)	6 (20.0)	
Spatial transcriptomics	3 (12.0)	0 (0.0)	3 (10.0)	
IHC	6 (24.0)	0 (0.0)	6 (20.0)	

Supplemental Table 1: Clinical characteristics of patients.

[#]Geldof, J et al. Classifying perianal fistulizing Crohn's disease: an expert consensus to guide decision-making in daily practice and clinical trials. Lancet Gastroenterol Hepatol. 2022 Jun;7(6):576-584.

*One patient with two samples at different clinical times was on infliximab at the time of one of the biopsies and was not on any biologics at the second biopsy.

[+]There were 25 samples for perianal Crohn’s disease for a total of 24 patients, and 30 samples were analyzed for 29 patients in the cryptoglandular fistula group (one patient in each group had two samples).
